# Effects of empagliflozin on serum uric acid level of patients with type 2 diabetes mellitus: a systematic review and meta‐analysis

**DOI:** 10.1186/s13098-023-01182-y

**Published:** 2023-10-16

**Authors:** Yinyuan You, Yu Zhao, Mujuan Chen, Ying Pan, Zhenhui Luo

**Affiliations:** Department of Pharmacy, Baoan Central Hospital of Shenzhen, No.3, Xiyuan Street, Bao’ an District, Shenzhen, Shenzhen, 518102 China

**Keywords:** Uric acid, Urate, Monosodium urate, Empagliflozin, Sodium–glucose transporter 2 inhibitors, SGLT-2 inhibitors, Gliflozins

## Abstract

**Background:**

Serum uric acid levels are higher in patients with type 2 diabetes and prediabetes compared to healthy individuals, and hyperuricemia causes a significant rate of complications and mortality through heart and kidney diseases. Accordingly, the present systematic review and meta-analysis aimed to investigate the effect of empagliflozin on serum uric acid levels.

**Materials and methods:**

Electronic databases, including PubMed, Scopus, Web of Science, Cochrane, and Google Scholar, were used to search papers until May 22, 2023. Data analysis was conducted by STATA Version 14, and P-value < 0.05 were considered statistically significant.

**Results:**

The results obtained from the combination of 12 studies with 7801 samples of diabetic patients indicated that in the empagliflozin group, the serum uric acid levels of the patients decreased ([standardized mean difference (SMD): − 1.97 (95%CI − 3.39, − 0.55)], Systolic blood pressure (SBP) [SMD: − 2.62 (95%CI − 3.87, − 1.37)] and diastolic blood pressure (DBP) [SMD: − 0.49 (95%CI − 0.68, − 0.29)]). On the other side, empagliflozin treatment did not affect the patients’ HbA1c levels ([SMD: − 2.85 (95%CI − 6.14, 0.45)], eGFR [SMD: 0.78 (95%CI − 0.63, 2.18)], creatinine [SMD:0.11 (95%CI − 0.10, 0.31)], LDL [SMD: 0.14 (95%CI − 0.43, 0.71)], and HDL [SMD:1.38 (95%CI − 0.22, 2.99)]). Compared with the placebo, empagliflozin was more effective in reducing the uric acid levels ([SMD: − 1.34 (95%CI − 2.05, − 0.63)], SBP [SMD: − 2.11 (95%CI − 3.89, − 0.33)], and HbA1c [SMD: − 1.04 (95%CI − 1.95, − 0.13)]). Moreover, compared with sitagliptin also, empagliflozin was more effective in reducing uric acid levels ([SMD: − 1 (95%CI − 1.78, − 0.22)], and creatinine [SMD: − 1.60 (95%CI − 2.28, − 0.92)]) and increasing eGFR levels [SMD: 0.99 (95%CI: 0.37, 1.62)] of the patients. Compared with dapagliflozin also, empagliflozin caused a reduction in eGFR level [SMD: − 0.45 (95%CI − 0.82, − 0.08)].

**Conclusion:**

Empagliflozin treatment was effective in controlling diabetic patients’ hyperuricemia and hypertension.

**Supplementary Information:**

The online version contains supplementary material available at 10.1186/s13098-023-01182-y.

## Introduction

The global prevalence of type 2 diabetes mellitus (T2DM) is steadily increasing, posing significant challenges to public health [1]. According to the International Diabetes Federation, the number of individuals with diabetes is expected to reach 552 million by 2030, resulting in substantial morbidity, mortality, and placing a burden on healthcare systems [2]. Given these projections, it is crucial to identify and mitigate risk factors associated with diabetes to prevent the development of complications. Hyperuricemia, a condition characterized by elevated serum uric acid levels, is a disorder of purine metabolism [3]. Serum Uric Acid (SUA) levels tend to be higher in individuals with T2DM and prediabetes compared to those without these conditions [4]. Hyperuricemia is influenced by various factors commonly found in diabetic patients, including increased body weight, waist circumference, dyslipidemia, sedentary lifestyle, hypertension, and insulin resistance [5, 6]. Studies indicated that increased SUA levels were positively associated with gout, kidney diseases, atherosclerosis, hypertension, stroke, and cardiovascular diseases [7, 8]. Hyperuricemia is also known to significantly increase the risk of mortality and complications, particularly in relation to kidney and cardiovascular diseases. Therefore, reducing SUA levels in patients with T2DM may potentially lower the occurrence of both minor and major complications [9, 10]. Sodium–glucose Cotransporter-2 (SGLT2) inhibitors are among the anti-diabetic medicines. SGLT2 reduces plasma glucose levels by decreasing renal glucose reabsorption and increasing urinary glucose excretion [11]. Previous studies indicated that, unlike allopurinol, SGLT2 inhibitors do not reduce uric acid production; instead, they increase its excretion rate [12].

Empagliflozin is one of the SGLT2 inhibitors, which may also affect serum lipid and uric acid levels and kidney function in addition to its antidiabetic effects [13–16]. The EMPA-REG OUTCOME trial indicated that empagliflozin reduced the risk of death from cardiovascular diseases, hospitalization due to heart failure, and progression of kidney failure to end‐stage kidney disease (ESKD) in patients with T2DM with increased cardiovascular disease risk [17, 18].

In this systematic review and meta-analysis, we aimed to investigate the relationship between empagliflozin treatment and serum uric acid (SUA) levels by combining the results of previous studies. While some studies have suggested a reduction in SUA levels with empagliflozin treatment [19, 20], others have found no statistically significant relationship [21, 22]. By conducting a systematic review and meta-analysis of randomized controlled trials, we sought to elucidate the role of empagliflozin on serum uric acid levels, providing up-to-date knowledge and answering specific research questions in a systematic and comprehensive manner [23, 24]. Therefore, in this systematic review and meta-analysis, we tried to investigate the relationship between the empagliflozin treatment and serum uric acid level by combining the results of the previous studies.

## Materials and methods

### Study design

The present study investigated the effect of empagliflozin treatment on SUA using the systematic review method and meta-analysis. This research is written according to Preferred reporting items for systematic review and meta-analysis [25], and its protocol was registered on the PROSPERO website (CRD42023438793).

### Data sources and searches

Electronic databases, including PubMed, Scopus, Web of Science, Cochrane, and Google Scholar, were used to search papers until May 22, 2023, without any time limit. MeSH keywords were used for the searches, and ‘AND, OR’ operators were used to combine the keywords. The keywords included: Uric Acid, Urate, Monosodium Urate, Empagliflozin, Sodium–Glucose Transporter 2 Inhibitors, SGLT-2 Inhibitors, and Gliflozins. Two authors searched the primary study sources to run the manual search. The search strategy of the PubMed database was as follows:

(“uric acid”[MeSH Terms] OR (“uric”[All Fields] AND “acid”[All Fields]) OR “uric acid”[All Fields] OR (“uratic”[All Fields] OR “uric acid”[MeSH Terms] OR (“uric”[All Fields] AND “acid”[All Fields]) OR “uric acid”[All Fields] OR “urate”[All Fields] OR “urates”[All Fields]) OR (“uric acid”[MeSH Terms] OR (“uric”[All Fields] AND “acid”[All Fields]) OR “uric acid”[All Fields] OR (“monosodium”[All Fields] AND “urate”[All Fields]) OR “monosodium urate”[All Fields])) AND (“empagliflozin”[Supplementary Concept] OR “empagliflozin”[All Fields] OR (“sodium glucose transporter 2 inhibitors”[Pharmacological Action] OR “sodium glucose transporter 2 inhibitors”[MeSH Terms] OR “sodium glucose transporter 2 inhibitors”[All Fields] OR “sodium glucose transporter 2 inhibitors”[All Fields]) OR (“sodium glucose transporter 2 inhibitors”[Pharmacological Action] OR “sodium glucose transporter 2 inhibitors”[MeSH Terms] OR “sodium glucose transporter 2 inhibitors”[All Fields] OR “SGLT 2 inhibitors”[All Fields]) OR “Gliflozine”[All Fields]) (Additional file 1: Table S1).

#### PICO components

Population: Studies related to patients with type 2 diabetes mellitus. Intervention: Empagliflozin treatment. Comparison: Placebo, dapagliflozin, and sitagliptin groups. Outcomes: The effect of empagliflozin treatment on Systolic blood pressure (SBP), diastolic blood pressure (DBP), high-density lipoprotein (HDL), low-density lipoprotein (LDL), creatinine, estimated glomerular filtration rate (eGFR), HbA1c, and SUA levels.

Inclusion criteria were RCTs and observational studies examining the effect of empagliflozin on serum uric acid in T2DM patients.

#### Exclusion criteria

Studies without accessible full texts, case-report studies, low-quality studies, letter-to-the-editor studies, replication studies, studies with incomplete data, studies that had investigated the effect of SGLT2 inhibitors treatment on SUA but did not separately report the effect of empagliflozin on SUA, and studies that had reported their findings qualitatively, were excluded.

#### Quality assessment

Two authors independently extracted relevant data using a predefined checklist. The quality of the observational studies was investigated using the STROBE checklist [26]. This checklist includes 22 questions, and the resulting score is a number between 0 and 44. The cut-off point of the STROBE checklist in the present study was a score of 16. The checklist presented by the Cochrane Institute was used to assess the clinical trials [27]. The checklist comprises seven questions, and each question investigates one of the major biases of the clinical trials and has three options to choose from (i.e., low-risk, high-risk, and unclear risk). Low-risk studies were included in the meta-analysis process.

#### Data extraction

A checklist was designed to extract data from the studies, and two authors separately extracted the data. The designed checklist included the name of the article author, publication year, mean age, dose and duration of empagliflozin treatment, study design, name of the country, sample size, comparison group, mean and standard deviation of SUA level, and other variables before and after the intervention.

#### Statistical analysis

All the studies included in the analysis incorporated both intervention and control groups, allowing for the computation of intra-group average difference and inter-group average difference indices. Statistical analysis involved utilizing the sample size, mean, and standard deviation of uric acid levels before and after the intervention in both groups. The standardized mean difference (SMD) index was then calculated. A value closer to zero on the SMD index suggests a weaker relationship, while a value closer to one or higher indicates a stronger relationship. Uric acid served as the primary outcome in all the studies, while SBP, HDL, LDL, creatinine, eGFR, HbA1c, UA, and DBP variables were considered secondary outcomes. However, the SMD index was employed for all of these variables. To assess heterogeneity, the Cochrane Q test was employed, and the I^2^ index was computed. Subgroup analysis and meta-regression were conducted to explore the sources of heterogeneity, and a Funnel plot was used to assess publication bias. The I^2^ index has three classifications: less than 25% indicates low heterogeneity, between 25 and 75% suggests moderate heterogeneity, and more than 75% signifies severe heterogeneity. The present study utilized a random effects model for data analysis. The analysis was performed using STATA 14 software, and a significance level of P < 0.05 was considered for the tests.

## Results

**Fig. 1:**
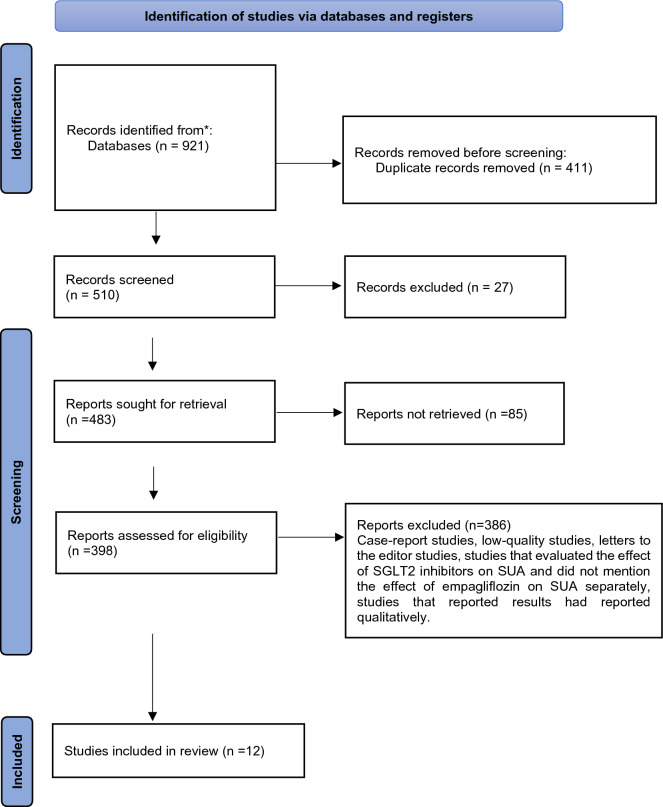
The process of entering the studies into the systematic review and meta-analysis

The searches in the mentioned databases yielded 921 articles. After reviewing the titles of the studies, 411 duplicate studies were excluded. The abstracts of the remaining 510 articles were reviewed, and 27 articles were removed due to the unavailability of the articles. From the remaining 483 articles, 85 titles were removed due to the lack of a complete dataset required for the analysis. Out of the remaining 398 articles, 386 were excluded according to the other exclusion criteria, and eventually, 12 articles with satisfactory quality were included in the meta-analysis process (Fig. 1).

Of the 12 investigated studies, two were observational, and ten were randomized controlled trials (RCTs). The mentioned studies investigated 7801 T2DM patients, and the mean ages of the individuals in the empagliflozin groups ranged between 51 to 78.4 years. On the other hand, the mean ages of the compared groups were between 47.8 and 78.4 years (Table 1).

**Table 1:** Characteristics of included studies

Author, year of publication	Country	Type of study	Sample size in empagliflozin group	Sample size in compare group	Mean age in empagliflozin group (year)	Mean age in compare group (year)	Duration of use	Dosage	Compare group
Schulze et al. 2022 [21]	Germany	RCT	30	29	72.9	76.05	4 weeks	25 mg	Placebo
Bogoviku et al. 2022 [19]	Germany	RCT	30	29	74.7	74.7	5 Day	25 mg	Placebo
Nesti et al. 2022 [28]	Italy	RCT	22	22	61.6	61.8	24 weeks	10 mg	Sitagliptin
Mozawa et al. 2021 [29]	Japan	RCT	46	50	63.9	64.6	24 weeks	10 mg	Placebo
Hiruma et al. 2021 [30]	Japan	RCT	21	21	52.8	47.8	12 weeks	10 mg	Sitagliptin
Okada et al. 2021 [20]	Japan	RCT	38	49	66	66	12 weeks	10 mg	Placebo
Okada et al. 2021 [20]	Japan	RCT	30	14	78.4	78.4	12 weeks	10 mg	Placebo
Shimizu et al. 2020 [31]	Japan	RCT	46	50	63.9	64.6	24 weeks	10 mg	Placebo
Gunhan et al. 2020 [32]	Turkey	Retrospective observational	70	49	56.46	56.46	24 weeks	NR	Dapagliflozin
Bosch et al. 2019 [33]	Germany	RCT	29	29	62	62	6-weeks	25 mg	Placebo
Inzucchi et al. 2018 [34]	USA	RCT	7020	NR	NR	NR	164 weeks	10 mg	Placebo
Inzucchi et al. 2018 [34]	USA	RCT	7020	NR	NR	NR	164 weeks	25 mg	Placebo
Yanai et al. 2017 [22]	Japan	Retrospective observational	15	20	51	52.3	12 weeks	25 mg	Dapagliflozin
Yanai et al. 2017 [22]	Japan	Retrospective observational	6	16	51	52.3	24 weeks	25 mg	Dapagliflozin
Heise et al. 2016 [35]	Germany	RCT	20	NR	56	NR	5 Day	25 mg	Placebo

*NR* not reported, *RCT* randomized clinical trial

**Fig. 2:**
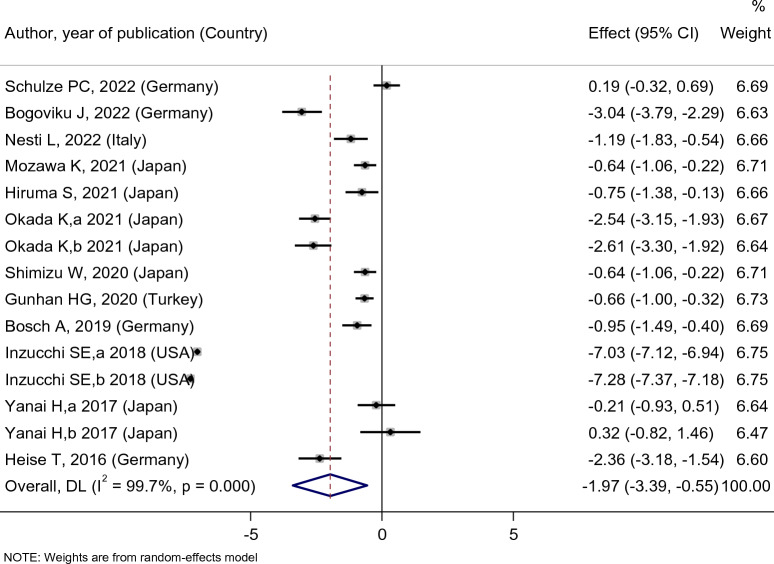
Forest plot showing the effect of empagliflozin on serum uric acid level

**Fig. 3:**
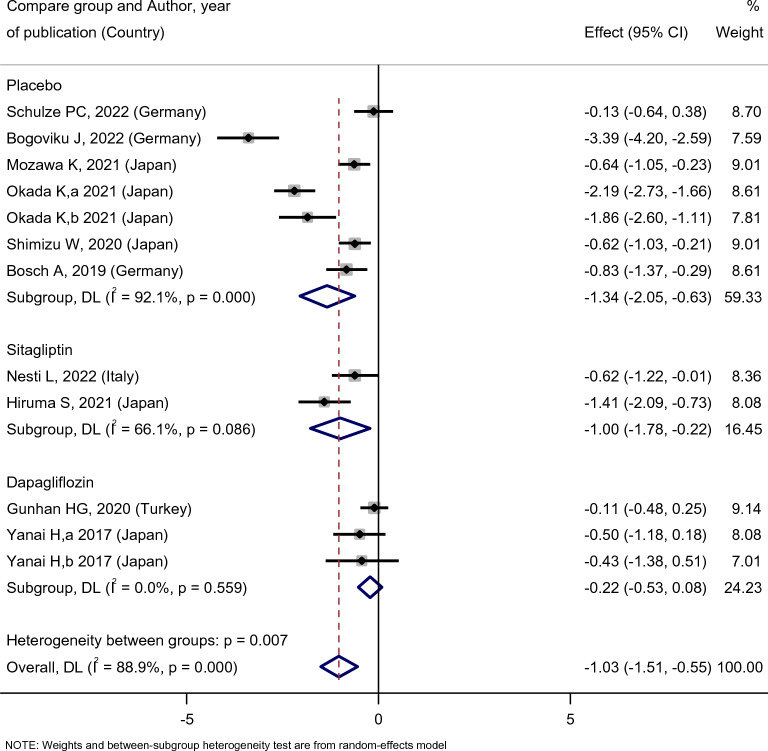
Standard mean difference and 95% confidence intervals for changes in serum uric acid level for empagliflozin compared to compare group

Empagliflozin treatment significantly reduced the serum uric acid level of diabetic patients [SMD: − 1.97 (95%CI − 3.39, − 0.55)] (Fig. 2). However, the changes in UA levels in patients of the empagliflozin group compared with the placebo, sitagliptin, and dapagliflozin groups were [SMD: − 1.34 (95%CI − 2.05, − 0.63)], [SMD: − 1 (95%CI − 1.78, − 0.22)], and [SMD: − 0.22 (95%CI − 0.53, 0.08)], respectively. The Results of this study indicated that empagliflozin was more effective in decreasing uric acid levels compared to placebo and sitagliptin. However, there was no statistically significant difference between the effects of empagliflozin and dapagliflozin on UA levels (Fig. 3).

In subgroup analysis, we showed that empagliflozin’s effect on UA level reduction in RCT studies was significant, while in observational studies was not. Moreover, the effect of empagliflozin treatment on UA levels of individuals who had used the medicine for 6, 12, 24, or 164 weeks was significant as it had reduced the UA level. However, we cannot conclude that longer treatment durations enhance the empagliflozin’s effect on UA level reduction. Regarding the dose, the effect of 10 and 25 mg empagliflozin doses on UA level reduction was not statistically significant. While in dose–response analysis, none of the doses (10 mg and 25 mg) were effective, the reasons for which the empagliflozin treatment was generally effective for UA level reduction (Fig. 2) was due to the facts: (A) the number of doses used per day is not known, and (B) the doses used by the study [27] was not reported, which caused it not to be a part of any of the subgroups (Table 2).

**Table 2:** The effect of empagliflozin on serum uric acid level based on different subgroups

Subgroups	SMD	Low limit	Up limit	P-value	I^2^ (%)
Type of study					
RCT	− 2.41	− 3.83	− 0.99	< 0.001	99.7
Observational	− 0.38	− 0.88	0.12	0.178	42.1
Time of treatment (weeks)					
≤ 6	− 1.52	− 2.93	− 0.10	< 0.001	95
12	− 1.53	− 2.72	− 0.35	< 0.001	92.3
24	− 0.67	− 0.93	− 0.42	0.248	26
164	− 7.15	− 7.39	− 6.92	< 0.001	92.8
Dosage (mg)					
10	− 2.20	− 5.22	0.81	< 0.001	99.8
25	− 1.91	− 5.26	1.44	< 0.001	99.7

*SMD* standardized mean difference

**Fig. 4:**
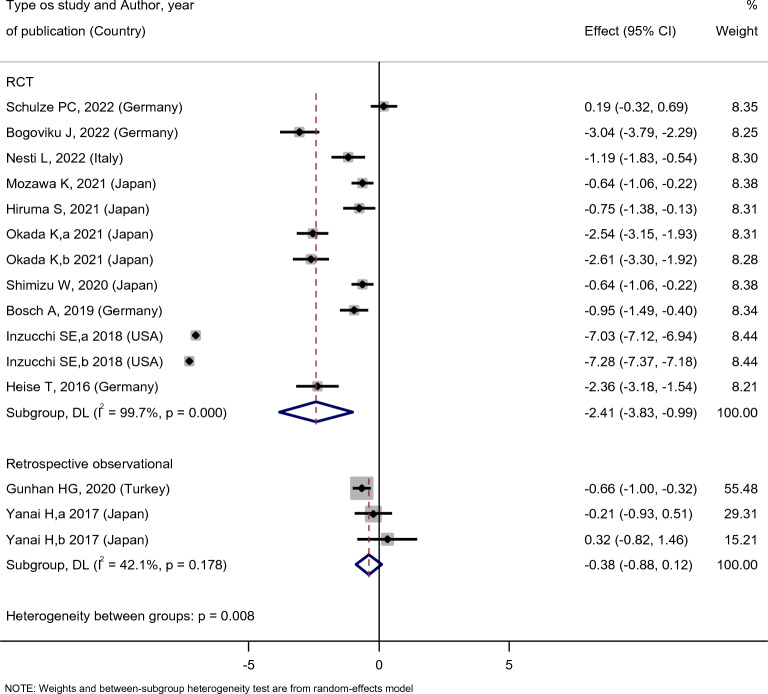
Subgroup Analysis of Randomized Controlled Trials and Observational Studies

In the subgroup analysis, the investigation was conducted based on the type of study, and we observed that in randomized controlled trials (RCTs), the effect of empagliflozin consumption on serum UA levels was [SMD: − 2.41 (95%CI − 3.83, − 0.99)], while in observational studies, it was [SMD: − 0.38 (95%CI − 0.88, 0.12)]. The effect of empagliflozin consumption on reducing serum UA levels was statistically significant in RCTs, but no statistically significant association was observed between empagliflozin consumption and serum UA levels in observational studies (Fig. 4).

**Fig. 5:**
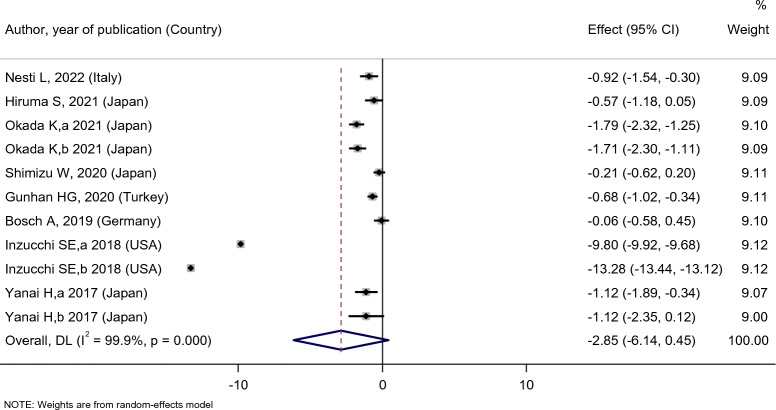
Forest plot showing the effect of empagliflozin on HBA1c level

**Fig. 6:**
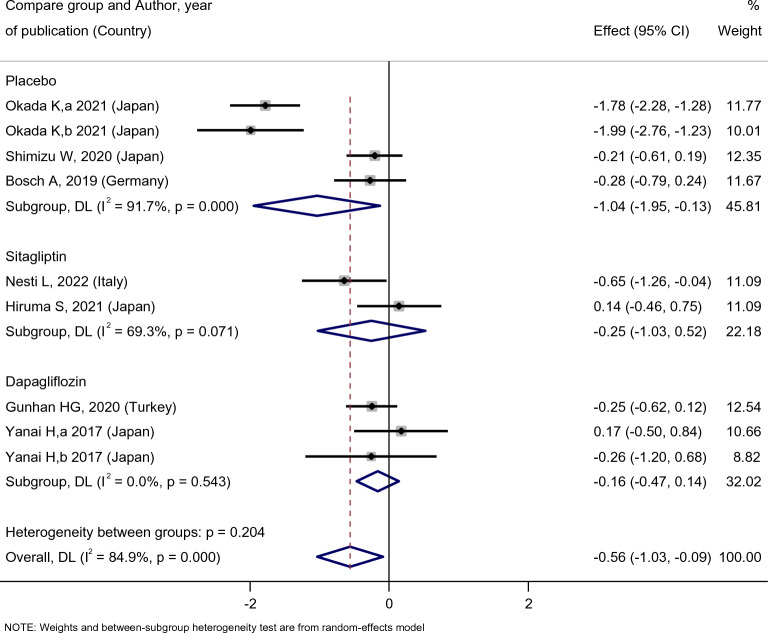
Standard mean difference and 95% confidence intervals for changes in serum HBA1c level for empagliflozin compared to compare group

Figure 5 indicated that empagliflozin treatment did not affect HbA1c level [SMD: − 2.85 (95%CI − 6.14, 0.45)]; however, compared with the placebo, empagliflozin significantly decreased the HbA1c level of the patients [SMD: − 1.04 (95%CI − 1.95, − 0.13)]. Nevertheless, compared with sitagliptin [SMD: − 0.25 (95%CI − 1.03, 0.52)] and dapagliflozin [SMD: − 0.16 (95%CI − 0.47, 0.14)], the effect of empagliflozin treatment on HbA1c was not statistically significant (Fig. 6).

**Fig. 7:**
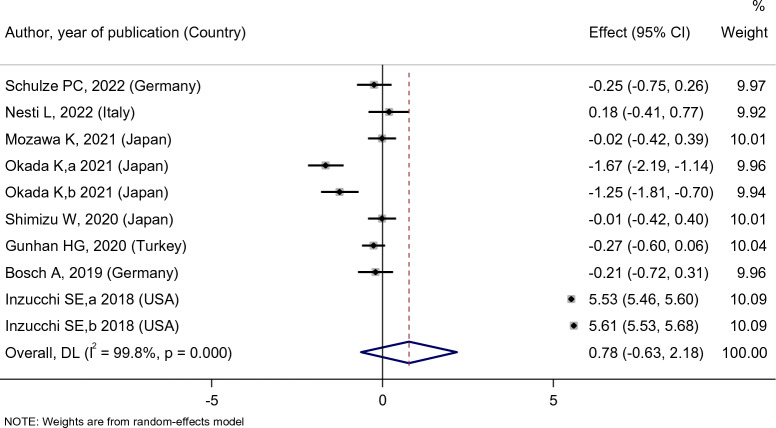
Forest plot showing the effect of empagliflozin on serum eGFR level

**Fig. 8:**
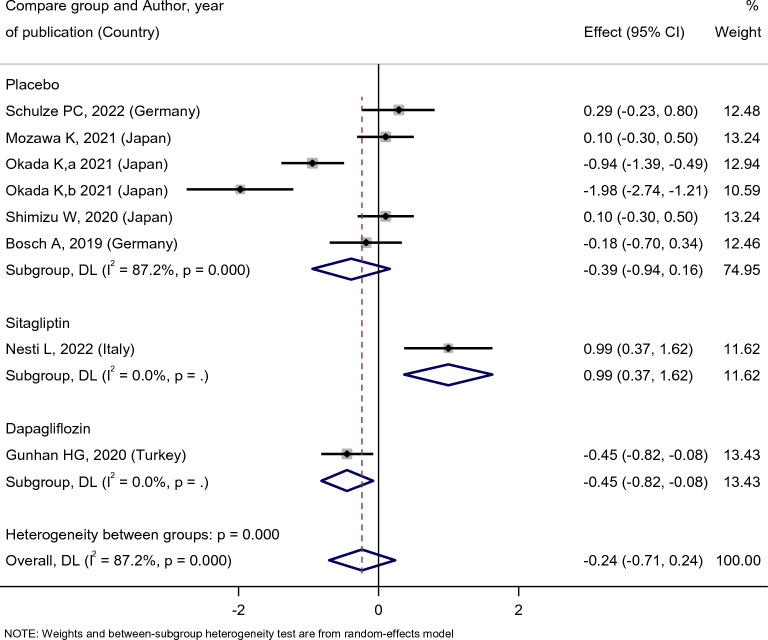
Standard mean difference and 95% confidence intervals for changes in serum eGFR level for empagliflozin compared to compare group

No statistically significant difference in the eGFR level of the empagliflozin group’s patients was observed after the empagliflozin treatment [SMD: 0.78 (95%CI − 0.63, 2.18)] (Fig. 7). Compared with placebo, empagliflozin did not affect the eGFR level of patients [SMD: 0.39 (95%CI − 0.94, 0.16)]. Compared with sitagliptin, however, empagliflozin treatment increased the eGFR level of patients [SMD: 0.99 (95%CI 0.37, 1.62)], and in comparison to dapagliflozin, empagliflozin treatment decreased the eGFR level [SMD: − 0.45 (95%CI − 0.82, − 0.08)]. However, it must be noted that there was only one study for dapagliflozin and sitagliptin groups, while five studies included placebo groups (Fig. 8).

**Fig. 9:**
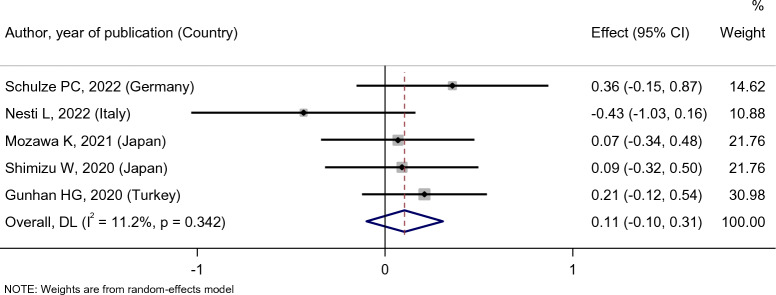
Forest plot showing the effect of empagliflozin on serum creatinine level

**Fig. 10:**
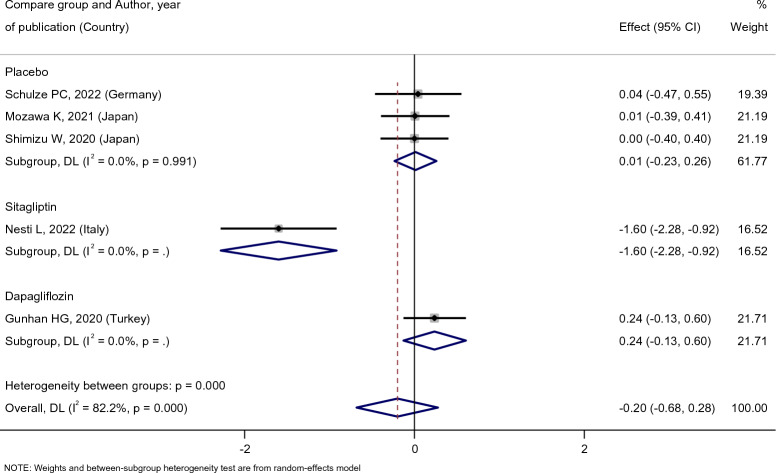
Standard mean difference and 95% confidence intervals for changes in serum creatinine level for empagliflozin compared to compare group

As Fig. 9 shows, the patient’s creatinine level did not change after empagliflozin treatment [SMD: 0.11 (95%CI − 0.10, 0.31)], and in Fig. 10 also the effect of empagliflozin on creatinine level compared with placebo and dapagliflozin were [SMD: 0.01 (95%CI − 0.23, 0.26)] and [SMD: 0.24 (95%CI − 0.13, 0.60)], respectively, and these relationships were not statistically significant. It must be noted that there was only one study for the placebo and dapagliflozin groups. On the other hand, observations indicated that compared with sitagliptin, empagliflozin was more effective in creatinine level reduction [SMD: − 1.60 (95%CI − 2.28, − 0.92)].

**Fig. 11:**
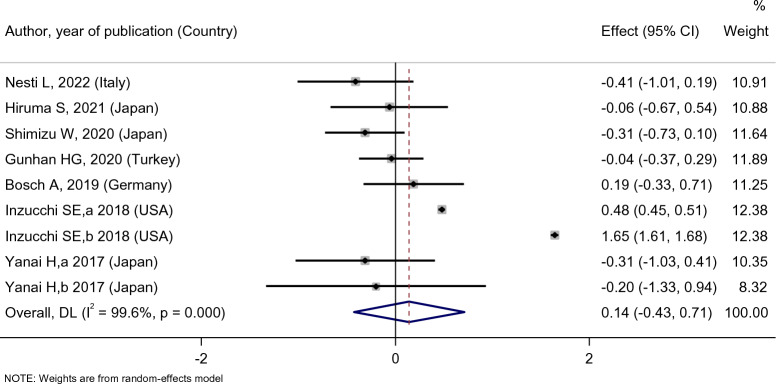
Forest plot showing the effect of empagliflozin on serum LDL level

**Fig. 12:**
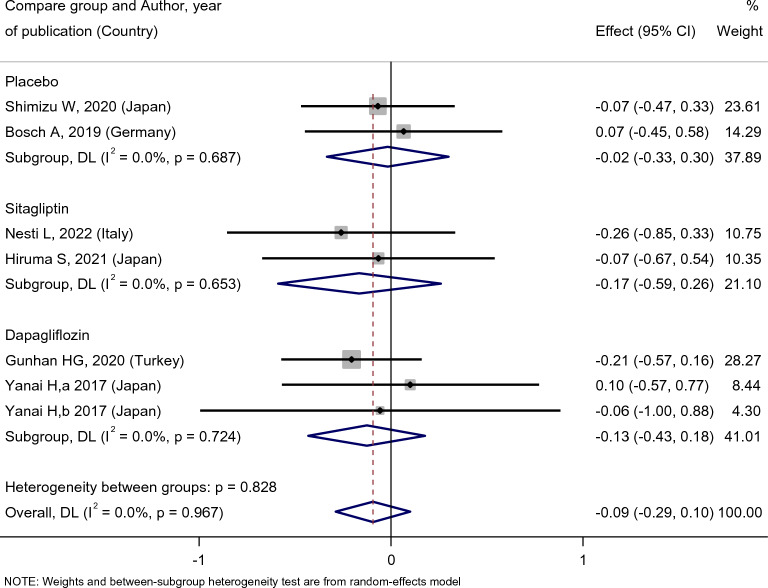
Standard mean difference and 95% confidence intervals for changes in serum LDL level for empagliflozin compared to compare group

While investigating the lipid profile, we concluded that empagliflozin treatment did not affect the patients’ LDL level [SMD: 0.14 (95%CI − 0.43, 0.71)] (Fig. 11). The result of group comparison also indicated that there was no statistically significant difference between the effects of empagliflozin on LDL level compared with the placebo group [SMD: − 0.02 (95%CI − 0.33, 0.30)], sitagliptin [SMD: − 0.17 (95%CI − 0.59, 0.26)], and dapagliflozin [SMD: − 0.13 (95%CI − 0.43, 0.18)] (Fig. 12).

**Fig. 13:**
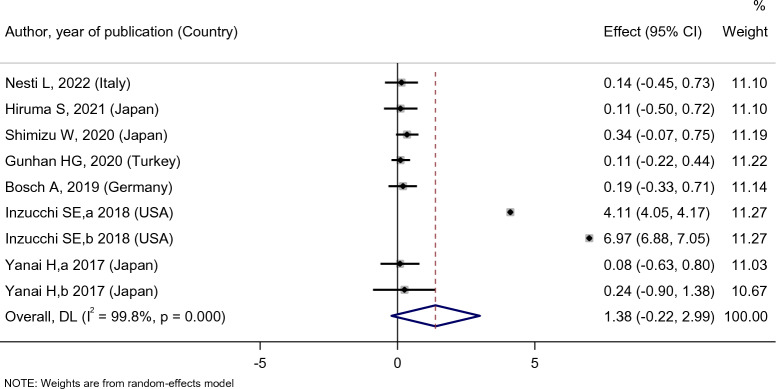
Forest plot showing the effect of empagliflozin on serum HDL level

**Fig. 14:**
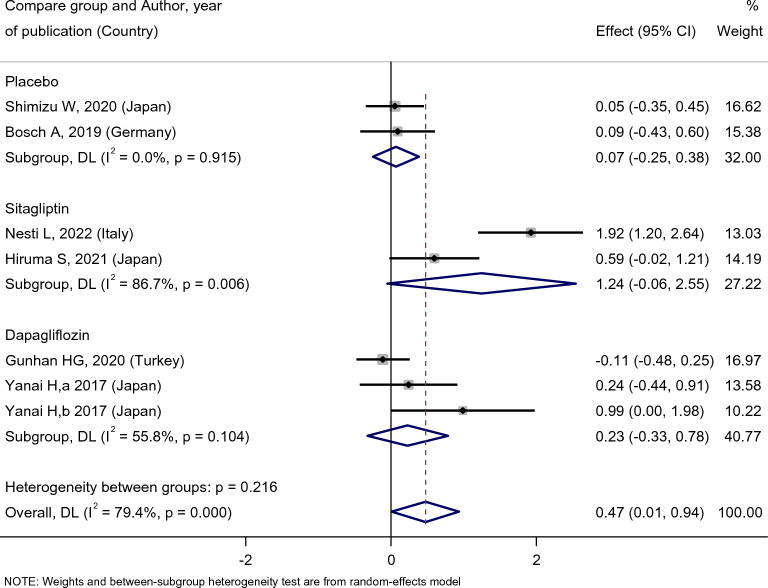
Standard mean difference and 95% confidence intervals for changes in serum HDL level for empagliflozin compared to compare group

Empagliflozin did not affect the patients’ HDL level [SMD: 1.38 (95%CI − 0.22, 2.99)] (Fig. 13), and there was no difference between the effect of empagliflozin treatment on HDL level compared with the placebo [SMD: 0.07 (95%CI − 0.25, 0.38)], sitagliptin [SMD:1.24 (95%CI − 0.06, 2.55)], and dapagliflozin [SMD: 0.23 (95%CI − 0.33, 0.78)] (Fig. 14).

**Fig. 15:**
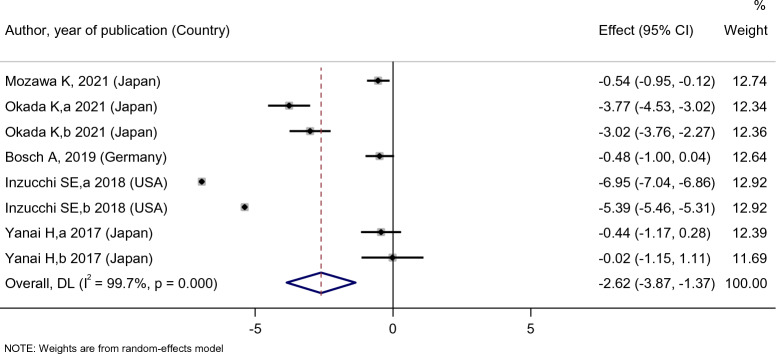
Forest plot showing the effect of empagliflozin on systolic blood pressure level

**Fig. 16:**
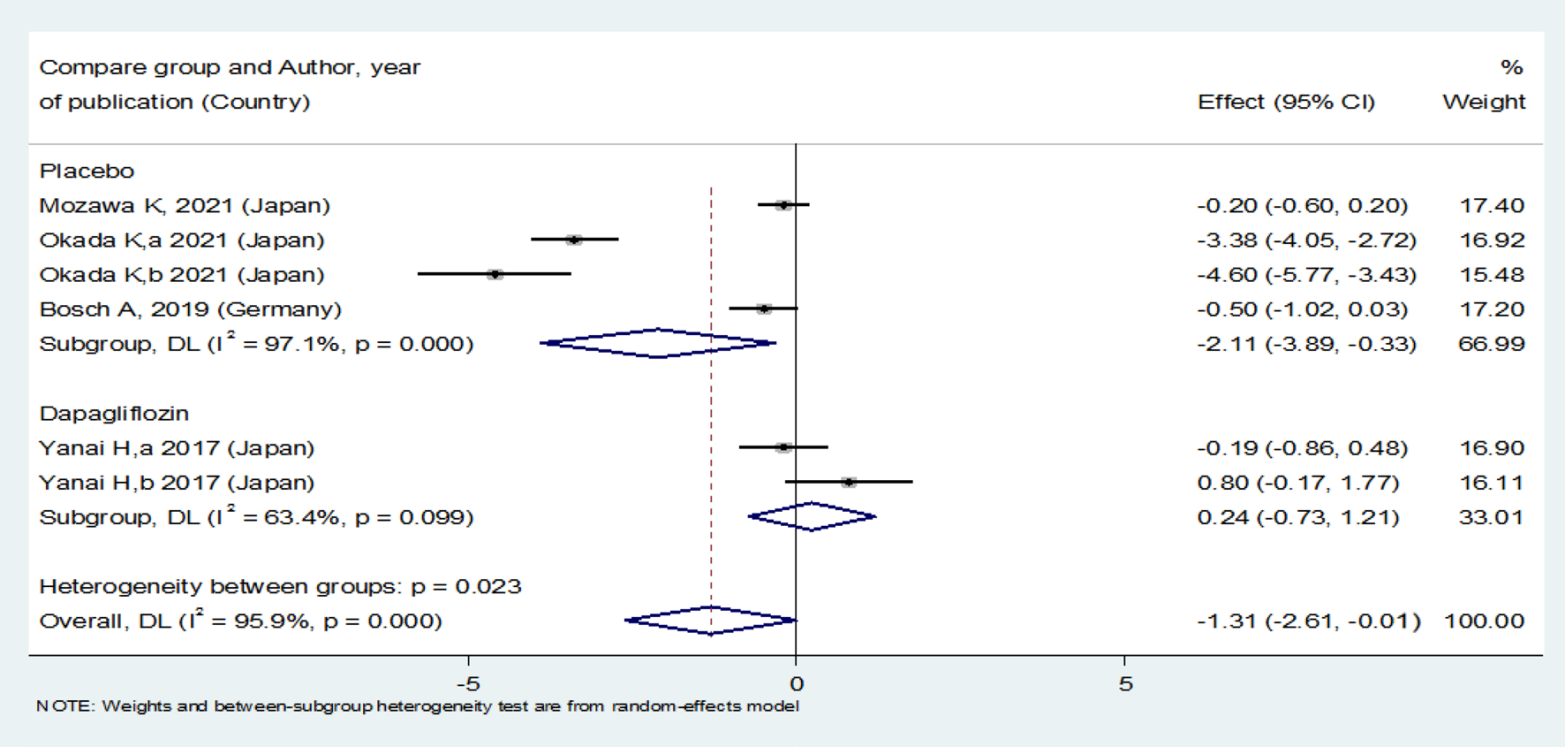
Standard mean difference and 95% confidence intervals for changes in systolic blood pressure level for empagliflozin compared to compare group

Empagliflozin treatment significantly reduced the patients’ SBP levels [SMD: − 2.62 (95%CI − 3.87, − 1.37)] (Fig. 15). On the other hand, empagliflozin was more effective in reducing SBP levels compared with the placebo [SMD: − 2.11 (95%CI − 3.89, − 0.33)]; however, there was no statistically significant difference between empagliflozin and dapagliflozin in reducing SBP level [SMD: 0.24 (95%CI − 0.73, 1.21)] (Fig. 16).

**Fig. 17:**
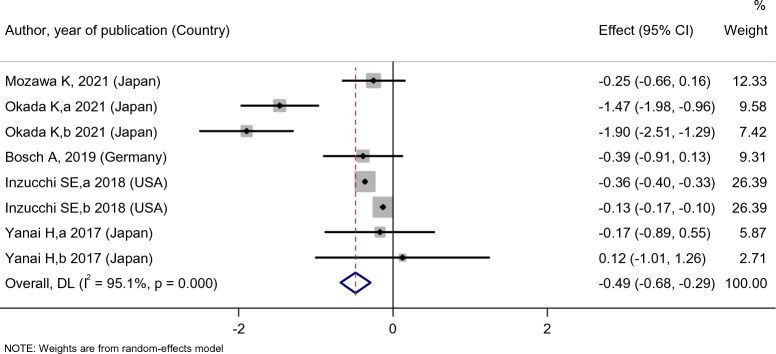
Forest plot showing the effect of empagliflozin on diastolic blood pressure level

**Fig. 18:**
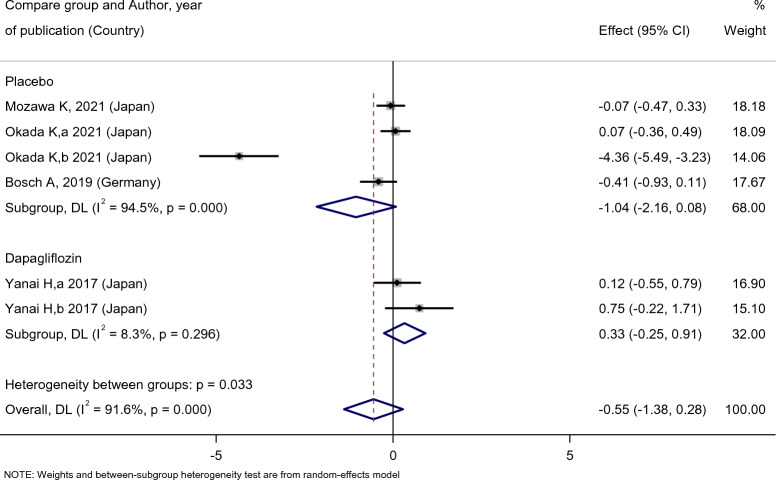
Standard mean difference and 95% confidence intervals for changes in diastolic blood pressure level for empagliflozin compared to compare group

Patients’ DBP levels slightly decreased after empagliflozin treatment [SMD: − 0.49 (95%CI − 0.68, − 0.29)] (Fig. 17). However, the effect of empagliflozin on DBP compared with the placebo [SMD: − 1.04 (95%CI − 2.16, 0.08)] and dapagliflozin [SMD: 0.33 (95%CI − 0.25, 0.91)] were not statistically significant (Fig. 18).

**Fig. 19:**
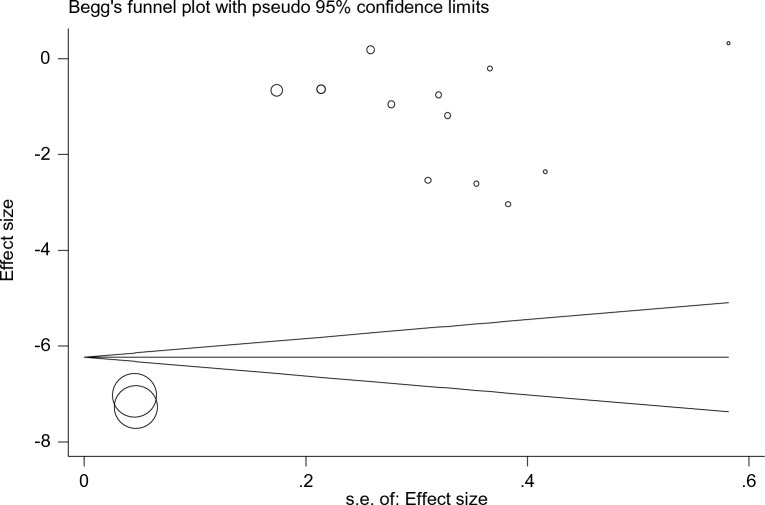
Funnel plot representing analysis of publication bias

Publication bias is statistically significant (P < 0.001) and indicates that articles declaring a negative or non-significant impact of empagliflozin on uric acid had no chance of being published and did not reach the dissemination stage (Fig. 19).

### Sensitivity analysis

**Fig. 20:**
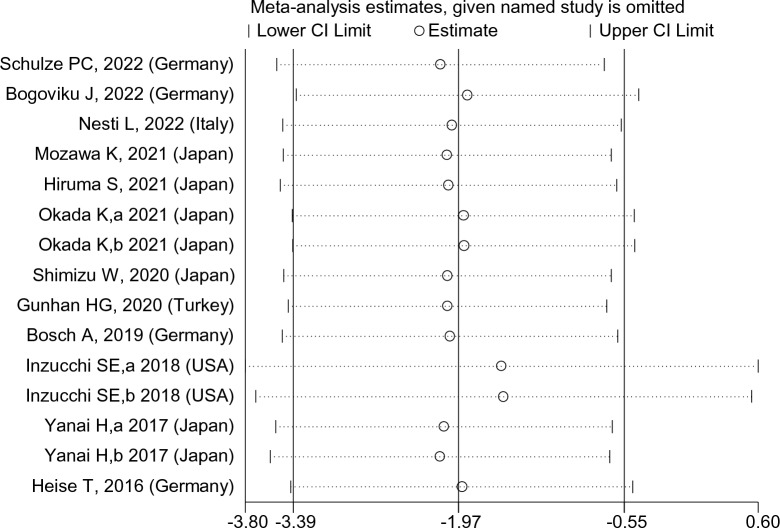
Impact of Excluding Influential Studies on Research Outcome

We examined the forest plots of both observational and clinical trials to investigate the impact of excluding individual studies on the overall findings of the research. As a result, we conducted a reanalysis to assess the influence of these studies on the outcomes. The most influential studies that significantly affected the final results of the research were the studies conducted by Yanai et al. [22] and Inzucchi et al. [34]. When the study by Yanai et al. [22] was excluded, the final outcome changed to an estimated standardized mean difference (SMD) of − 2.09 (95% confidence interval [CI] − 3.53, − 0.65). Similarly, when the study by Inzucchi et al. [34] was removed, the projected final outcome was an SMD of − 1.58 (95% CI − 3.70, 0.54) (Fig. 20).

## Discussion

Our meta-analysis indicated that empagliflozin treatment was effective in lowering high blood pressure and hyperuricemia in diabetic patients. The SGLT2 inhibitors decrease UA levels by increasing urinary excretion and possibly through decreasing reactive oxygen species, which reduce the activity of the xanthine oxidase enzyme [36–38]. In the following sections, we will discuss similar studies.

Our findings demonstrate a significant reduction in hyperuricemia among diabetic patients through the effective use of Empagliflozin treatment. In a recent meta-analysis conducted by Hu et al. [39] on randomized controlled trials involving patients with type 2 diabetes mellitus (T2DM) in Asia, it was observed that SGLT-2 inhibitors, including Empagliflozin, significantly lowered serum uric acid (SUA) levels compared to the control group [MD = − 0.965, 95% CI (− 1.029, − 0.901)]. Similarly, other available meta-analyses have reported that SGLT2 inhibitors, such as Canagliflozin [WMD − 37.02 μmol/L, 95% CI (− 38.41, − 35.63)], Dapagliflozin [MD − 38.05 μmol/L, 95% CI (− 44.47, − 31.62)], and Empagliflozin [WMD − 42.07 μmol/L, 95% CI (− 46.27, − 37.86)], significantly reduce SUA levels in comparison to placebo [40]. Furthermore, Ferreira et al. [41] conducted a meta-analysis on diabetic patients and found that Empagliflozin treatment exhibited a reduction in SUA levels compared to placebo [mean treatment difference = 0.37 (95% CI 0.42, 0.31) mg/dL]. The mentioned results were consistent with the findings of the present meta-analysis and indicated that empagliflozin in doses of 10 mg and 25 mg is effective in treating hyperuricemia in T2DM patients. These findings collectively support the effectiveness of Empagliflozin in lowering SUA levels in individuals with T2DM.

In a study by Hussain et al. [42] which aimed to compare the effect of dapagliflozin and empagliflozin on SUA level of T2DM patients against the traditional oral antihyperglycemic drugs after four weeks of medication, the mean SUA level of SGLT2 inhibitors group reduced from 7.5 ± 2.5 mg/dl to 6.3 ± 0.8 mg/dl, but the mean SUA level of the comparison group reduced from 7.1 ± 1.8 mg/dl to 6.8 ± 2.2 mg/dl. The mentioned study showed that SGLT2 inhibitors were more effective in reducing SUA levels than the comparison group [42]. Based on the results of a study by Zhao et al. [36], every SGLT2 inhibitor significantly reduced SUA levels compared with the control group [(Total WMD) − 37.73 μmol/L, 95% CI (− 40.51, − 34.95)]. Empagliflozin treatment led to a more acceptable reduction in SUA level [WMD − 45.83 μmol/L, 95% CI (− 53.03, − 38.63)] [36]. Similarly, in the present meta-analysis, the effect of empagliflozin treatment on reducing the SUA levels compared with the base mode was statistically significant in every duration of the medication (i.e., 6 weeks, 12 weeks, 24 weeks, or 164 weeks).

Findings of a study by Zhao et al. on 5781 type 2 diabetes mellitus patients indicated that empagliflozin reduced the SBP, DBP, UA, HbA1C, fasting plasma glucose, and body weight of T2DM patients. However, regarding the GFR, there was no difference between empagliflozin and placebo [43]. In the present study, the SBP, DBP, and UA levels decreased compared with the base values after the empagliflozin treatment, but there was no statistically significant change in GFR; hence, we confirmed the result of the previous study. However, the findings regarding the HbA1C of the present study were not consistent with the previous study.

Investigations on the effects of empagliflozin treatment on metabolic parameters, cardiovascular risk factors, and anthropometric measurements of T2DM patients indicated that after six months of empagliflozin treatment, body weight, SBP, LDL, fasting plasma glucose, and HbA1c significantly reduced compared with the base values. However, the estimated GFR, serum creatinine, UA, total cholesterol, triglyceride, and HDL levels had no significant change [44]. Our findings confirmed some of the resulting parameters of the mentioned study, but the findings were not consistent in several parameters. A meta-analysis of studies comparing the effects of empagliflozin before and after treatment found that empagliflozin did not significantly affect HbA1c, eGFR, creatinine, LDL, or HDL levels. However, empagliflozin was found to significantly reduce blood pressure. Our findings provide valuable insights into the multifaceted mechanisms that contribute to the antihypertensive properties of SGLT2 inhibitors. Our study contributes to the understanding of the mechanisms by which SGLT2 inhibitors lower blood pressure in patients with type 2 diabetes mellitus. The observed effects involve diuresis, natriuresis, improvement in endothelial function, attenuation of arterial stiffness, and potential weight loss. Further investigations are needed to fully unravel the intricate interplay between these mechanisms and to determine the long-term impact of SGLT2 inhibitors on blood pressure control. These findings have important clinical implications, suggesting the potential utility of SGLT2 inhibitors as adjunctive therapy for hypertension management in patients with type 2 diabetes [14, 43].

Combining metformin and empagliflozin has potential for managing hyperuricemia and reducing uric acid levels in diabetes [45, 46]. Empagliflozin increases uric acid excretion and inhibits reabsorption, while metformin improves insulin sensitivity and modifies purine metabolism. Synergistic effects are possible [19, 34, 45]. However, some studies show no significant changes [47]. Limited data availability prevented our analysis. Further research is needed for consistent and long-term effects. Clinical implications in diabetes management should be investigated.

The meta-analysis of 12 studies in the present study indicates that empagliflozin effectively reduces serum uric acid levels in patients with type 2 diabetes mellitus (T2DM). However, it is important to address the issues of heterogeneity and publication bias. The included studies exhibited substantial heterogeneity, as evidenced by an I^2^ value of 99.7%, indicating significant variation in methodologies, populations, and other factors that could impact the results. Furthermore, the exclusion of influential studies by Yanai et al. [22] and Inzucchi et al. [34] resulted in a significant reduction in uric acid levels. After excluding Yanai et al. [22], the estimated standardized mean difference (SMD) for serum uric acid levels was − 2.09 (95% CI − 3.53, − 0.65). Similarly, removing the study by Inzucchi et al. [34] yielded a projected SMD of − 1.58 (95% CI − 3.70, 0.54). These findings indicate the potential influence of these studies on the overall outcomes. Furthermore, the study’s results suggest the presence of publication bias, with a statistically significant result (P < 0.001). This indicates that articles reporting negative or non-significant effects of empagliflozin on uric acid levels may have faced challenges in publication and dissemination, potentially impacting the interpretation of the findings. Given these considerations, it is crucial to acknowledge the heterogeneity among the studies and the potential influence of publication bias. Therefore, further research and additional data are necessary to draw a valid and comprehensive conclusion regarding the effectiveness of empagliflozin in reducing uric acid levels in patients with T2DM.

The main strength of our study was the rigorous exclusion of other influential risk factors that could impact diabetes outcomes, including hypertension, HbA1c, eGFR, creatinine, LDL, and HDL levels. Additionally, we specifically focused on the effects of a single drug, Empagliflozin, which is known to affect SUA levels, and we minimized heterogeneity to enhance the reliability of our findings. However, our study had certain limitations. The differences in the characteristics of the groups investigated resulted in a smaller number of included studies and, consequently, smaller sample sizes in each group (placebo, dapagliflozin, and sitagliptin). We also faced limitations in accessing the full text of several studies, which may have impacted our ability to fully analyze the data. In addition, we couldn’t present results by age groups because the mean ages reported in the studies we reviewed were similar and overlapped. Furthermore, since the reviewed studies didn’t provide gender-specific data, our study didn’t explore the association between patients’ gender and the impact of empagliflozin on their SUA levels. The distribution of studies across different countries was uneven, with a significant number of studies conducted in countries such as the United States, while some countries under investigation did not have published studies available. Another limitation was the lack of discussion regarding the relationship between patients’ sex and the effect of empagliflozin on their SUA levels, as the included studies did not provide separate data based on patients’ sex. These limitations should be acknowledged when interpreting the findings of our study. Future research should aim to address these limitations, including a more balanced distribution of studies across different countries, consideration of age group differences, and the inclusion of data related to patients’ sex, in order to provide a more comprehensive understanding of the relationship between empagliflozin and SUA levels.

## Conclusion

In conclusion, empagliflozin treatment decreased serum uric acid levels and blood pressure in patients with type 2 diabetes, but did not affect glycemic control or kidney function. Empagliflozin was more effective than placebo and some other SGLT2 inhibitors in reducing uric acid levels. Further large RCTs are required to confirm these findings.

### Supplementary Information

**Additional file 1: Table S1.** MeSH terms with Boolean operators.

## Data Availability

The corresponding author can provide access to the datasets used in this study upon a reasonable request.
