# Supplementary material for: Effects of empagliflozin on serum uric acid level of patients with type 2 diabetes mellitus: a systematic review and meta‐analysis
Source: Diabetol Metab Syndr. 2023 Oct 16;15:202. doi: 10.1186/s13098-023-01182-y (PMC10577917; doi:10.1186/s13098-023-01182-y)
Supplement: Supplementary file 1 — Additional file 1: Table S1. MeSH terms with Boolean operators. [file 13098_2023_1182_MOESM1_ESM.docx]

***Search strategy in databases:***

***PubMed***

(Uric Acid OR Urate OR Monosodium Urate) AND (Empagliflozin OR Sodium-Glucose Transporter 2 Inhibitors OR SGLT-2 Inhibitors OR Gliflozine): 209 results

***Web of Sciences***

[Uric Acid OR Urate OR Monosodium Urate (All Fields) and Empagliflozin OR Sodium-Glucose Transporter 2 Inhibitors OR SGLT-2 Inhibitors OR Gliflozine (All Fields)](https://www2.wosgs.ir/wos/woscc/summary/10bc2b87-2fc6-4aef-a699-068f2db5daf5-92404f3a/relevance/1): 250 results

***Cochrane***

## Uric Acid OR Urate OR Monosodium Urate in Title Abstract Keyword AND Empagliflozin OR Sodium-Glucose Transporter 2 Inhibitors OR SGLT-2 Inhibitors OR Gliflozine in Title Abstract Keyword: 162 results
